# Chronic Heart Disease after Treatment of Oral Acute Chagas Disease

**DOI:** 10.5935/abc.20160115

**Published:** 2016-08

**Authors:** Andrei Fornanciari Antunes, Simão Gonçalves Maduro, Bruna Valessa Moutinho Pereira, Maria das Graças Vale Barbosa, Jorge Augusto de Oliveira Guerra, João Marcos Bemfica Barbosa Ferreira

**Affiliations:** 1 Hospital Universitário Francisca Mendes, Manaus, AM - Brazil; 2 Universidade Estadual do Amazonas (UEA), Manaus, AM - Brazil; 3 Fundação de Medicina Tropical Heitor Vieira Dourado, Manaus, AM - Brazil

**Keywords:** Chagas Disease, Chagas Cardiomyopathy, Trypanosoma cruzi

## Abstract

We describe the recurrence of cardiac abnormalities in a patient treated during the acute phase of Chagas disease after outpatient follow-up of 5 years.

## Introduction

Chagas disease, described more than 100 years ago by Carlos Chagas, is considered by the World Health Organization (WHO) as one of the most neglected tropical diseases worldwide. Prevalent in developing countries, it has a major social and economic impact in many regions of Latin America. The natural course of the disease, initially characterized by an acute phase presenting as non-specific oligosymptomatic febrile illness, followed by a chronic phase with long, latent evolution, may hinder the timely diagnosis to appropriate treatment.^1,2^

Since the 1990s, the Amazon region has experienced an increased incidence of isolated cases or small outbreaks of acute chagas disease (ACD), with most cases resulting from oral transmission by ingestion of food containing vector remains or their waste infected with *Trypanosoma cruzi*.^2-4^

In a previous publication, we described the cardiac involvement in five patients with acute Chagas disease treated with benznidazole.^4^

In this report, we describe one of these cases in detail, which, during the long-term follow-up, showed cardiac involvement recurrence after 5 years of treatment, despite negative serological and parasitological tests.

## Case Report

**Figure 1:**
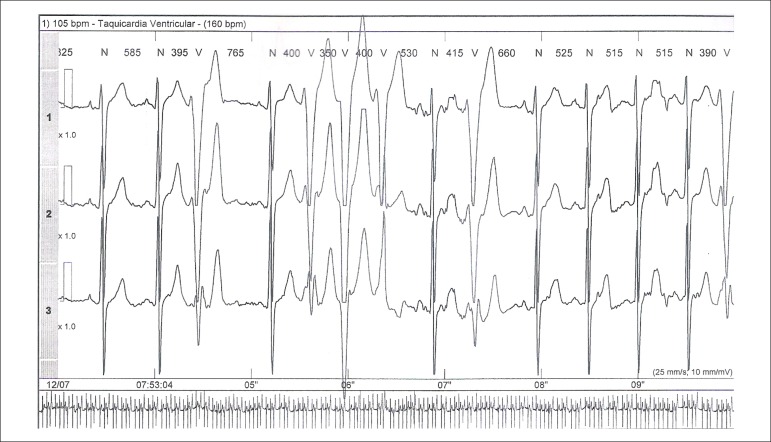
ECG recording of nonsustained ventricular tachycardia episode on Holter.

**Figure 2:**
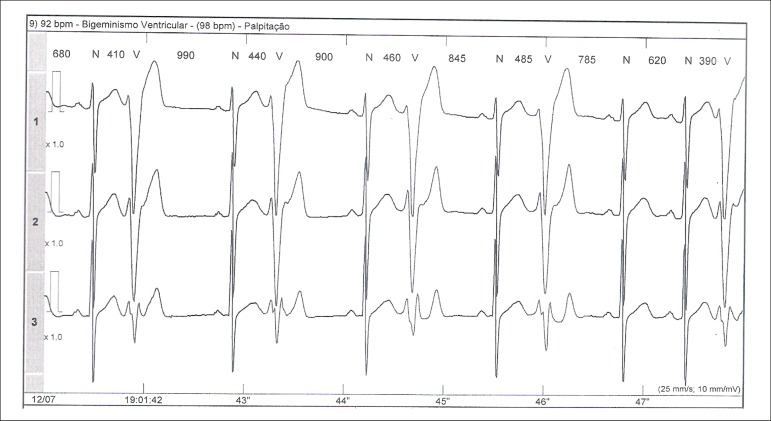
ECG recording of ventricular bigeminy on Holter.

JANF, a male individual from the rural area of the city of Manaus (state of Amazonas, Brazil), was 15 years old in 2007 when he presented with a clinical picture of ACD related to oral transmission caused by acai juice intake. At the time, he developed palpitations, chest pain and dyspnea on moderate exertion. The electrocardiogram at rest showed frequent ventricular extrasystoles and the echocardiogram showed mild left ventricular dysfunction with ejection fraction of 50%. He was treated for heart failure with captopril, carvedilol and furosemide, as well as for Chagas disease, with benznidazole for 60 days. When the treatment was finished, the patient became asymptomatic and heart tests were normal. He also had negative serology and parasitological tests for Chagas disease. After remaining asymptomatic for five years, the patient once again started to have tachycardia. The electrocardiogram showed isolated ventricular ectopic activity, whereas the echocardiography and cardiac MRI results were normal. The outpatient electrocardiographic recording (Holter) showed frequent monomorphic ventricular ectopic activity, episodes of ventricular bigeminy and frequent episodes of nonsustained ventricular tachycardia (Figures 1 and 2). Immunological and parasitological tests for Chagas disease (thick film for *T. cruzi* identification, xenodiagnosis and PCR) were negative, ruling out the presence of the acute phase of Chagas disease reactivation. Antiarrhythmic treatment with amiodarone (200 mg/day) was initiated, with symptom improvement and electrocardiographic parameter normalization.

## Discussion

During the acute phase of Chagas disease, most patients have a benign prognosis, and complete symptom remission occurs between 60 and 90 days, regardless of the therapeutic intervention.^3,5^ The goal of the disease treatment in its acute phase is to eradicate the parasite, fight the signs and symptoms and prevent progression to the chronic form of the disease, which, in turn, results in great morbidity and mortality over the years. There have been reports showing the disease acquired by oral transmission has more a severe clinical course and a higher mortality rate.^6^

This patient showed good response to benznidazole therapy and, at the end of the treatment, had complete regression of cardiac abnormalities, as well as negative serological and parasitological tests.

However, after five years, he showed cardiac symptom recurrence with complaints of tachycardia and the ECG disclosed the presence of ventricular arrhythmia. Transthoracic echocardiography and cardiac MRI did not show any morphological and/or functional alterations. The normal imaging test results suggested a probable chagasic etiology, as they did not show any alterations suggestive of other differential diagnoses, such as arrhythmogenic right ventricular dysplasia.

The main hypotheses for the genesis of ventricular arrhythmias in this patient would be the presence of small areas of interstitial fibrosis/scarring, autonomic dysfunction or microcirculation disorder. These alterations have been observed in patients with Chagas disease and may lead to electric decoupling, preventing adequate stimulus conduction and resulting in potential reentrant circuits, which generate arrhythmias.^7^

The absence of detectable fibrosis in the cardiac MRI does not completely rule out the possibility of small areas of myocardial interstitial fibrosis. A previous study in patients with another type of heart disease showed a sensitivity of only 74% of the MRI to detect focal myocardial fibrosis when compared with histopathology.^8^ Additionally, another study showed that in approximately 21% of patients with positive serology for Chagas disease and evidence of ventricular arrhythmias, there is no detectable myocardial fibrosis on the MRI.^9^

The autonomic nervous system was evaluated by heart rate variability in the time domain, which is a validated method for this analysis and the results were considered normal in relation to the reference values of the European and American guidelines (SDNN = 161 ms, SDANN = 144 ms, pNN50 = 21% and RMSSD = 44 ms).^10^ However, autonomic function has a complex mechanism and several methods can be used in its study and there is no gold standard test for its assessment.^10^ The exercise test using amiodarone showed chronotropic deficit and no induction of significant ventricular arrhythmias.

Reports in the literature of patients treated in the acute phase of Chagas disease with long-term follow-up showed persistent ECG and/or echocardiographic alterations in spite of treatment. It is not known whether these alterations correspond to the chronic phase of Chagas disease or to an acute involvement sequel in a parasite-free patient.^5^

The present case is noteworthy, as it refers to a patient treated in the acute phase of Chagas disease with cardiac involvement, with normalization of symptoms and heart tests and who, at the end of a 5-year follow-up, experienced recurrence of cardiac ventricular arrhythmia, in the absence of disease-reactivation criteria, but showing evolution to the arrhythmogenic chronic form of the disease. This abnormality can result in significant morbidity and mortality, with high risk of sudden cardiac death and long-term severe ventricular dysfunction.

## Conclusion

The evolution to the chronic form of Chagas' disease is an undesirable event. In order to prevent this outcome, the adequate treatment of the disease during its acute phase is essential. The long-term follow-up is also necessary, considering the physiopathological complexity of this disease, making it difficult to establish accurate criteria for the cure, in spite of the normalization of all currently available laboratory tests.
